# Dynamic Changes in the Potential Suitable Habitat of *Caragana korshinskii* Under Climate Change Based on a Biomod2 Ensemble Model

**DOI:** 10.3390/plants15132001

**Published:** 2026-06-28

**Authors:** Xuhu Wang, Furong Niu

**Affiliations:** College of Forestry, Gansu Agricultural University, Lanzhou 730070, China

**Keywords:** desertification, *Caragana korshinskii*, Biomod2 ensemble model, climate change, potential suitable habitat

## Abstract

Projecting the spatiotemporal dynamics of the potential distribution of dominant species under climate change is essential for desertification control and vegetation restoration in drylands. Here, we modeled the current (1970–2000) and future (2080–2100) suitable habitats of *Caragana korshinskii* Kom, an ecologically important shrub species in northwestern China, by constructing an ensemble of eight species distribution models on the Biomod2 platform using three CMIP6 Shared Socioeconomic Pathways (SSP126, SSP370, SSP585) and 40 environmental variables representing climate, soil, topography and drought conditions. Key environmental drivers were identified through variable importance ranking and response curves, while area changes, spatial patterns, and centroid shifts in suitable habitats were quantified. The ensemble model demonstrated good to excellent predictive performance (mean AUC > 0.9, mean TSS > 0.5). Soil base saturation (t-bs) and soil moisture contributed the most (>38%), highlighting the dominant role of edaphic factors. The current total suitable habitat of *C. korshinskii* is approximately 182.2 × 10^4^ km^2^, with all future scenarios projecting a consistent decline. Under SSP585, habitat loss reached 9.8% with contraction (30.5 × 10^4^ km^2^) far exceeding expansion (12.6 × 10^4^ km^2^). The distribution centroid shifted markedly eastward with a minor southward fluctuation, establishing the Ordos–Bayannur region as a stable core habitat. Overall, our findings suggest that the distribution of C. *korshinskii* is strongly constrained by edaphic and moisture conditions, and future contraction of marginal habitats may compromise ecosystem services.

## 1. Introduction

Global warming has become a major driving force altering the structure and function of terrestrial ecosystems [1]. Under high-emission scenarios, the temperature is projected to increase by 3.3–5.7 °C by 2100 [2]. Rising temperatures and shifts in precipitation regimes are profoundly affecting the geographic distributions of species, leading to latitudinal and elevational shifts in biomes [3,4]. Arid and semi-arid regions cover approximately 41% of the Earth’s land surface and support about 38% of the global human population; their ecosystems are particularly fragile, and the water-limited productivity makes these regions far more sensitive to climatic fluctuations than humid regions [5,6]. Desertification involves the rapid alteration of soil properties, vegetation patterns, and hydrological conditions, profoundly affecting inland regions and posing significant challenges to human societies [7,8]. Vegetation in arid zones performs critical ecological functions such as sustaining livestock and wildlife and preventing soil desertification [9]. In these water-limited ecosystems, native dominant shrubs often function as ecosystem engineers, playing a disproportionately important role in maintaining community stability, conserving soil and water, providing wildlife habitat, and regulating nutrient cycling [5,6]. Understanding the potential distribution dynamics of these keystone species under climate change is therefore critical for predicting dryland ecosystem trajectories and formulating proactive restoration strategies. Consequently, the response of dryland vegetation to climate change has become a global research hotspot. Desertified areas in northern China serve as a critical component of the national ecological security barrier, where vegetation degradation and desertification processes coexist. There is an urgent need to clarify the responses of key native species to future climate change in order to formulate proactive ecological restoration strategies [10].

When evaluating species suitable for afforestation and carbon sequestration in arid regions, drought tolerance and dominant species status are usually considered [11,12]. In afforestation practices in arid and semi-arid regions, dominant species can mitigate the negative impacts of reduced precipitation by protecting water resources [13]. *Caragana korshinskii* Kom. (Fabaceae) is a perennial shrub widely distributed in the Loess Plateau, the Ordos Plateau, the Alxa Plateau, and the eastern Hexi Corridor of China [14]. This species possesses remarkable drought tolerance, nutrient-poor soil endurance, and nitrogen-fixing ability. It is one of the most extensively planted shrub species in the Three-North Shelterbelt Program and plays a central role in windbreak and sand-fixation, soil and water conservation, and soil improvement [15]. *C. korshinskii* fixes atmospheric nitrogen through symbiosis with rhizobia, enabling its establishment on extremely infertile soils, and its deep root system can access deep soil water, demonstrating a conservative water-use strategy [16]. Additionally, *C. korshinskii* is regarded as an effective non-conventional feed resource for grazing livestock during winter and spring when other forages are scarce [17], and has long been used as animal feed or an important protein source in ruminant diets [18].

Species distribution models (SDMs) are key technical tools for predicting the potential suitable habitats of species and their responses to climate change. Ranging from early bioclimatic envelope models (e.g., BIOCLIM) to machine-learning methods (e.g., MaxEnt, Random Forest), SDMs statistically or mechanistically link species occurrence points with environmental variables. They have been widely applied in conservation biology, invasion biology, and global change ecology [19,20]. However, single models often produce divergent predictions due to inherent algorithmic biases, especially when extrapolating to novel climates. To address this, ensemble models that combine predictions from multiple algorithms have been developed. By integrating the strengths of different modeling approaches, ensemble models significantly reduce predictive uncertainty and improve robustness compared to any single model [21,22]. The Biomod2 platform integrates multiple algorithms (e.g., GLM, GAM, MARS, Random Forest, MaxEnt) using a committee averaging strategy, which can effectively reduce model uncertainty and improve prediction robustness [21,23]. Despite its ecological and practical importance, previous modeling studies on *C. korshinskii* have often relied on single-algorithm models, been restricted to specific regions (e.g., the Loess Plateau), and employed limited climatic variables or a single future scenario, lacking a comprehensive, large-scale assessment of distributional dynamics and the dominant role of soil properties under a robust ensemble framework [24]. This limits our ability to make reliable predictions about its future range shifts. Furthermore, the CMIP6 Shared Socioeconomic Pathways (SSPs) provide plausible greenhouse gas emission trajectories under different development pathways, making it possible to assess the ecological consequences of different policy choices [25]. These scenarios range from the low-emission SSP126 (a sustainability pathway with radiative forcing of 2.6 W/m^2^ by 2100) to the high-emission SSP370 (a regional rivalry pathway with 7.0 W/m^2^), and the very high-emission SSP585 (a fossil-fueled development pathway with 8.5 W/m^2^) [26]. Therefore, focusing on *C. korshinskii*, this study employed the Biomod2 ensemble modeling approach based on 40 environmental variables (covering climate, soil, topography, and drought indices) under three future emission scenarios (SSP126, SSP370, SSP585) for the period 2080–2100. This end-of-century time slice is widely used in CMIP6 projections to capture the maximum divergence in climate conditions among emission pathways [25] and provides a sufficiently long window for long-lived perennial shrubs such as *C. korshinskii* to exhibit distributional responses to climate change. We aimed to answer the following three scientific questions: (1) What are the key environmental factors constraining the distribution of *C. korshinskii*? (2) How will the total suitable habitat area and its internal hierarchical structure change under different emission scenarios? (3) In which direction will the distribution centroid shift, and which regions will become future stable habitats? The results will provide spatially explicit decision support for the conservation and restoration of important native shrubs in arid and semi-arid regions.

## 2. Results

### 2.1. Model Accuracy and Variable Contribution

**Figure 1:**
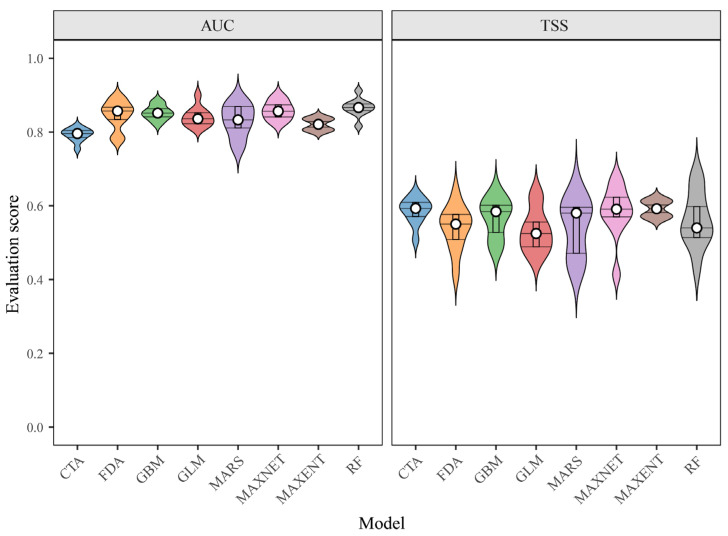
Accuracy evaluation of the eight individual species distribution model algorithms and the ensemble model. The x-axis displays the algorithm abbreviations: CTA, Classification Tree Analysis; FDA, Flexible Discriminant Analysis; GBM, Generalized Boosting Model; GLM, generalized linear model; MARS, Multivariate Adaptive Regression Splines; MAXENT, maximum entropy; MAXNET, maximum entropy (GLM); RF, Random Forest. Panel the left graph presents area under the receiver operating characteristic curve (AUC) values, and the right graph shows the true skill statistic (TSS) values.

**Figure 2:**
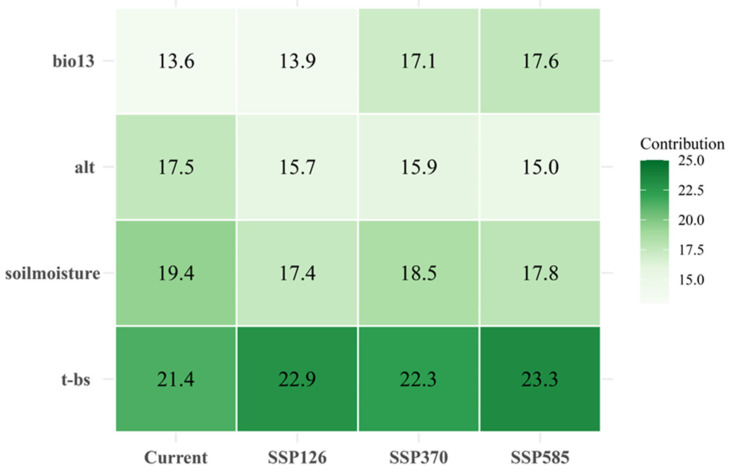
Contribution rates of the four most influential environmental variables under current and future climate scenarios. The x-axis displays four climate scenarios: Current (1970–2000), SSP126, SSP370, and SSP585 (2080–2100). The y-axis shows variable importance (%). Variable abbreviations: bio13, precipitation of wettest month; alt, altitude; soilmoisture, topsoil moisture content; t-bs, topsoil base saturation. Note: After collinearity filtering, 11 environmental variables were retained for ensemble modeling (see Table 1). The four variables shown here together account for approximately 65–70% of the total contribution; the remaining ~30–35% is distributed among the other seven variables (bio1, bio4, bio12, bio15, t-gravel, t-oc, t-ph-H_2_O). The sum of the contribution rates of all 10 variables equals 100%.

**Figure 3:**
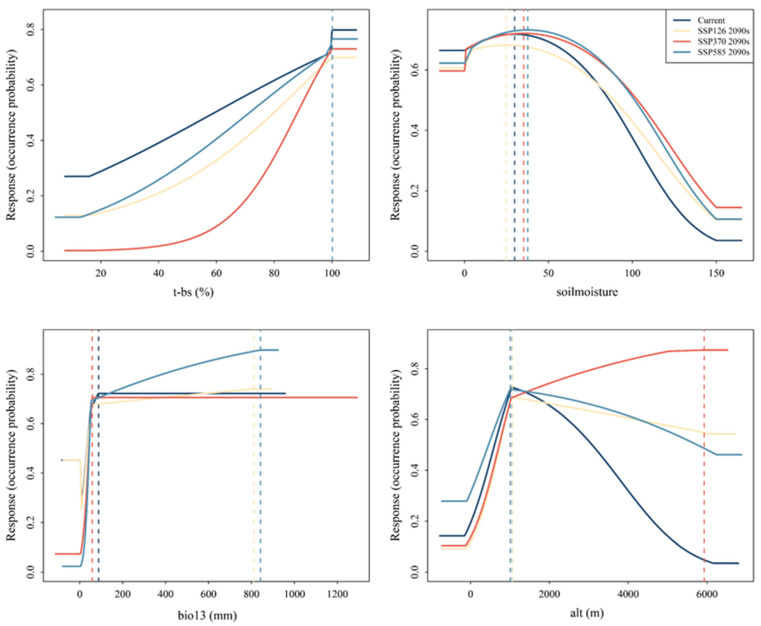
Response curves of dominant environmental factors. The vertical dashed lines indicate species optimal environmental values in the 2090s under SSP126 (yellow, low emission), SSP370 (red, medium emission) and SSP585 (light blue, high emission) scenarios. Dark blue solid curves correspond to current climate, and the dark blue dashed line at curve peaks represents the current optimal environmental threshold.

The Biomod2 ensemble model achieved mean AUC values ranging from 0.79 to 0.86 and mean TSS values from 0.52 to 0.57, indicating that all individual models had good predictive ability, and weighted integration further reduced prediction variance (Figure 1). The final ensemble model, obtained through TSS-weighted committee averaging, demonstrated good overall performance with an AUC of 0.92 and a TSS of 0.75. Variable importance analysis (Figure 2) showed that soil base saturation (t-bs) was the primary factor in all scenarios, with contribution rates of 21.4–23.3%, followed by soil moisture (soilmoisture) (17.4–19.4%); the combined contribution of these two variables exceeded 38%. They were followed by altitude (alt, 15.0–17.5%) and precipitation of the wettest month (bio13, 13.6–17.6%). The contribution rates of annual mean temperature (bio1) and annual precipitation (bio12), traditionally considered as dominant for dryland vegetation, were less than 10% and 15%, respectively, underscoring the decisive role of soil properties in shaping the distribution of this species. Response curves showed (Figure 3) that the occurrence probability of t-bs stabilized within the 60–85% interval and remained at a high plateau as the value approached 100%, slightly declining above 98%; soil moisture exhibited a left-skewed unimodal distribution, with an optimal range of approximately 15–25%, and suitability declined rapidly above 35%; suitability decreased continuously above a 1500 m altitude and was nearly zero above 2500 m; the response to bio13 was relatively flat without a distinct optimal peak. These response patterns provide important ecological insights into the environmental constraints on *C. korshinskii*. The near-monotonic increase in suitability with base saturation confirms its strong calcicole affinity, likely reflecting the physiological requirement of its rhizobial symbionts for calcium ions as nitrogenase cofactors [27] and the mitigation of aluminum and manganese toxicity in high base saturation soils [28]. The left-skewed unimodal response to soil moisture, with a sharp decline above 35%, indicates that while *C. korshinskii* requires adequate soil water for its establishment, it is highly sensitive to waterlogging, consistent with its conservative isohydric regulation strategy and the risk of root hypoxia under excessive moisture [16]. The continuous decline in suitability above 1500 m altitude reflects the constraints of low temperature, shortened growing season, and intense freeze–thaw cycles on seedling recruitment and growth at high elevations [29,30]. The flat response to precipitation of the wettest month (bio13) suggests broad tolerance to short-term extreme rainfall intensity, further underscoring that soil properties and drainage capacity, rather than precipitation extremes alone, are the primary determinants of habitat suitability for this species [31].

**Table 1:** Environmental variables initially considered for modeling the potential distribution of *C. korshinskii*. The table lists 40 variables across five categories: climatic factors (19 bioclimatic variables from WorldClim 2.1), soil factors (16 topsoil properties from HWSD), topographic factors (3 variables from SRTM DEM), and drought factors (2 variables from CGIAR-CSI). Variable codes, full descriptions, and measurement units are provided. The prefix “t-” in soil variable codes denotes topsoil properties (0–30 cm depth). Variables marked with an asterisk (*) are those retained after Pearson correlation filtering (|r| ≤ 0.7) and used in the final ensemble modeling. A total of 11 variables were retained after collinearity analysis.

Type	Variable	Description and Unit	Type	Variable	Description and Unit
Climatic factors	bio1 *	Annual mean temperature (°C)	Soil factors	t-ece	Topsoil electrical conductivity (dS/m)
	bio2	Mean diurnal range (°C)		t-texture	Topsoil texture class
	bio3	Isothermality		t-clay	Topsoil clay content (% wt)
	bio4 *	Temperature seasonality		t-cec-soil	Topsoil cation exchange capacity (cmol/kg)
	bio5	Max temperature of warmest month (°C)		t-cec-clay	Topsoil CEC of clay fraction (cmol/kg)
	bio6	Min temperature of coldest month (°C)		t-CaSO_4_	Topsoil sulfate content (% weight)
	bio7	Temperature annual range (°C)		t-CaCO_3_	Topsoil carbonate content (% weight)
	bio8	Mean temperature of wettest quarter (°C)		t-bs *	Topsoil base saturation (%)
	bio9	Mean temperature of driest quarter (°C)		t-gravel *	Topsoil gravel content (% vol.)
	bio10	Mean temperature of warmest quarter (°C)		t-oc *	Topsoil organic carbon content (% weight)
	bio11	Mean temperature of coldest quarter (°C)		t-ph-H_2_O *	Topsoil pH (−log[H^+^])
	bio12 *	Annual precipitation (mm)		t-ref-bulk	Topsoil bulk density (kg/m^3^)
	bio13 *	Precipitation of wettest month (mm)		t-sand	Topsoil sand content (% wt.)
	bio14	Precipitation of driest month (mm)		t-silt	Topsoil silt content (% wt.)
	bio15 *	Precipitation seasonality (coefficient of variation)		t-teb	Topsoil total exchangeable bases (cmol/kg)
	bio16	Precipitation of wettest quarter (mm)		t-usda-tex-clay	Topsoil USDA texture classification (name)
	bio17	Precipitation of driest quarter (mm)	Topographic factors	aspect	Aspect (°)
	bio18	Precipitation of warmest quarter (mm)		Alt *	Altitude (m)
	bio19	Precipitation of coldest quarter (mm)		slope	Slope (°)
Soil factors	t-esp	Topsoil exchangeable sodium percentage (%)	Drought factors	ai	Aridity index (%)
	soil moisture *	Topsoil moisture content		et0	Potential evapotranspiration (mm)

Note: The prefix “t-” indicates that the soil property refers to the topsoil layer (0–30 cm depth).

### 2.2. Spatial Pattern Change in Suitable Habitat

**Figure 4:**
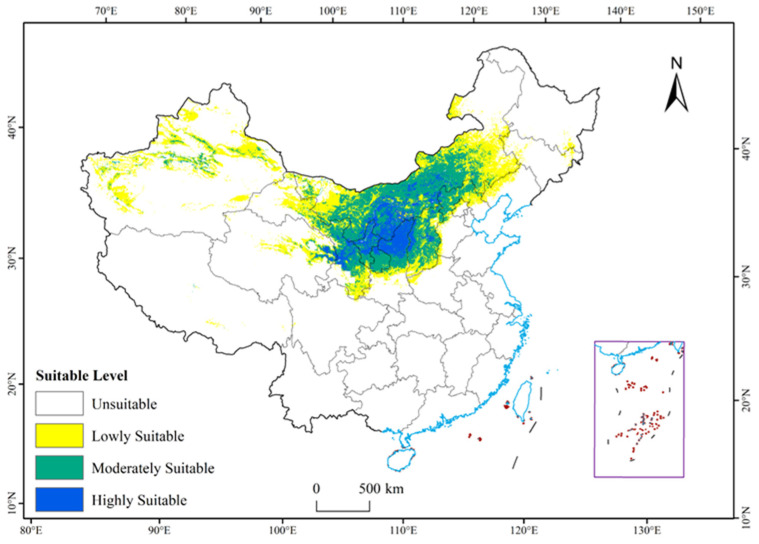
Current potential distribution of *Caragana korshinskii.* The color scheme represents four suitability classes: blue, high suitability; green, moderate suitability; yellow, low suitability; white, unsuitable.

**Figure 5:**
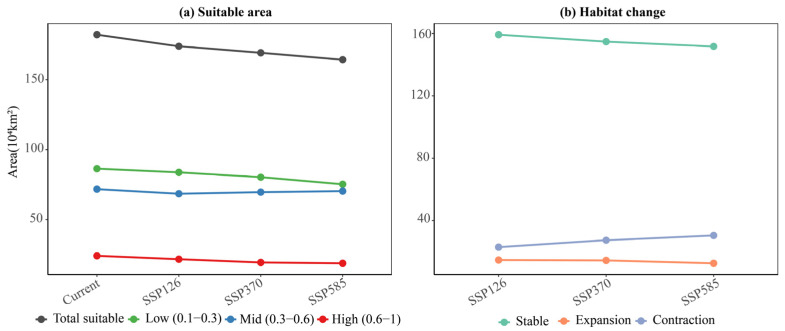
Area changes in suitable habitat for *Caragana korshinskii* under current and future scenarios. The figure consists of two panels: (**a**) suitable habitat area under current (1970–2000) and future (2080–2100) climate scenarios across four suitability classes; (**b**) spatial dynamics of habitat change relative to the current baseline, classified into Stable (suitable under both current and future conditions), Expansion (unsuitable under current but suitable under future conditions), and Contraction (suitable under current but unsuitable under future conditions). Change values are calculated as differences relative to the current baseline period (1970–2000).

**Figure 6:**
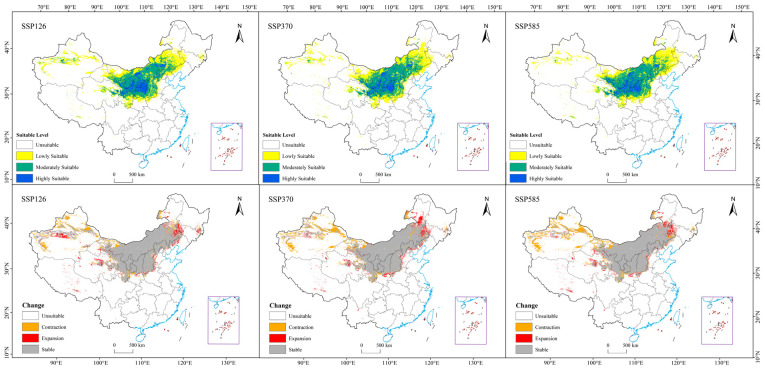
Current and future distribution dynamics of *Caragana korshinskii.* The figure consists of six panels: the upper three panels show the potential suitable habitat distribution under three future climate scenarios (SSP126, SSP370, and SSP585, 2080–2100), respectively; the lower three panels show the corresponding spatial dynamics of habitat change relative to the current baseline (1970–2000). Color scheme for suitability maps (**upper panels**): blue, high suitability; green, moderate suitability; yellow, low suitability; white, unsuitable. Color scheme for change maps (**lower panels**): orange, contraction (suitable under current but unsuitable under future); red, expansion (unsuitable under current but suitable under future); gray, stable (suitable under both current and future conditions).

Under the current climate scenario, the potential suitable habitat of *C. korshinskii* exhibited distinct geographic gradients (Figure 4). The overall distribution was dominated by the Ordos Plateau and the Loess Plateau, extending northeastward to the eastern Hetao Plain south of the Yin Mountains, and southwestward covering the southern edge of the Alxa Plateau and entering the eastern Hexi Corridor. Highly suitable areas occurred as patches scattered around Ordos City, the Ningxia Plain, and the hilly loess region of northern Shaanxi; moderately suitable areas formed a relatively continuous band surrounding the highly suitable areas; lowly suitable areas further transitioned to the desert steppe in the northwest, even touching the southeastern edge of the Badain Jaran Desert. The current total suitable area was 182.2 × 10^4^ km^2^, with highly suitable areas accounting for only 24.1 × 10^4^ km^2^ (Figure 5). Under future climate scenarios, the distribution pattern showed an overall trend of northward contraction and northeastward migration (Figure 6). Under SSP126, the total suitable area decreased to 173.9 × 10^4^ km^2^ (−4.5%), with retreat mainly occurring in marginal low-suitability zones on the western side of the Alxa Plateau and the western Loess Plateau. Under SSP370, the area further decreased to 169.3 × 10^4^ km^2^ (−7.1%), with the highly suitable area shrinking dramatically (−19.6%) and increased fragmentation. Under the high-emission SSP585 scenario, the total area dropped to 164.4 × 10^4^ km^2^ (−9.8%), with the highly suitable area reduced to only 18.7 × 10^4^ km^2^ (−22.0%); the moderate and low suitability zones also exhibited multiple isolated patches, indicating severe compression and erosion of core ecological space.

### 2.3. Spatial Migration Pattern of Habitats

By overlaying the current and future binary suitability maps, the spatiotemporal dynamics of *C. korshinskii* habitats were quantified (Figure 5 and Figure 6). The results revealed a severe asymmetric change pattern: the contraction area was significantly larger than the expansion area under all scenarios. Specifically, under SSP126, the contraction area reached 23.0 × 10^4^ km^2^, while the expansion area was only 14.7 × 10^4^ km^2^, a ratio of approximately 1.6. This imbalance intensified with increasing emission levels. Under SSP585, the contraction area expanded sharply to 30.5 × 10^4^ km^2^, whereas the expansion area was only 12.6 × 10^4^ km^2^, with the ratio rising to approximately 2.4. This means that under the extreme warming pathway, for every 1 km^2^ of newly gained potential habitat, *C. korshinskii* would simultaneously lose 2.4 km^2^ of its original habitat.

**Figure 7:**
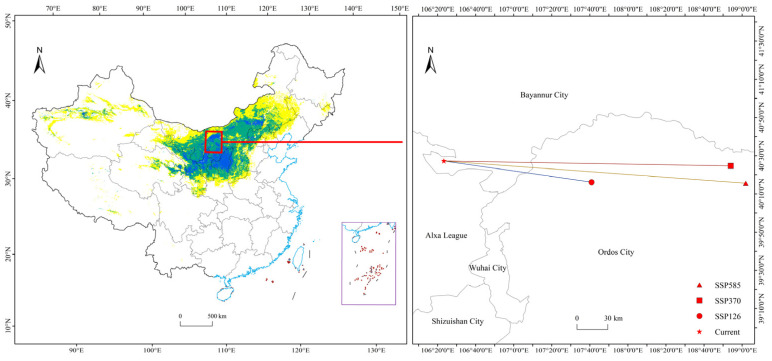
Distribution centroids of *Caragana korshinskii* under current and different future scenarios. The color represents the occurrence suitability probability of the species, which is consistent with the color representation in Figure 4.

Spatially, the expansion area mainly occurred as narrow, scattered strips on the northeastern edge of the current distribution range, i.e., north of the Kubuqi Desert and south of the Yin Mountains, pointing toward the northern part of Ordos City and the southern part of Bayannur City, suggesting that these areas may become more suitable in the future (Figure 7). The stable area (suitable under both current and future conditions) still dominated in terms of area (151.7–159.3 × 10^4^ km^2^) and was also concentrated in the northeastern region centered on Ordos–Bayannur. In contrast, the contraction area occurred as large, contiguous patches along the southwestern and northwestern boundaries of the distribution range, covering most of the Alxa Plateau and the outer edges of the western Loess Plateau. These regions represent the front line of habitat retreat for *C. korshinskii*.

### 2.4. Centroid Response of the Distribution

Consistent with the spatial changes, the geographic distribution centroid of the suitable habitat of *C. korshinskii* showed a pronounced eastward shift with a minor southward fluctuation (Figure 7). The current centroid is located at 106.39° E, 40.45° N. Under SSP126, the centroid shifted to 107.68° E, 40.27° N (southward by ~0.18°); under SSP370, it moved to 108.90° E, 40.41° N (southward by ~0.04°); and under SSP585, the centroid reached 109.03° E, 40.26° N (southward by ~0.19°). Overall, the centroid shifted eastward by more than 2.6° of longitude, whereas the latitudinal change remained within a narrow band of approximately 0.2° south of the current centroid. The current centroid is situated in the central-eastern Alxa Plateau, an arid region characterized by mean annual precipitation of 100–200 mm, desert steppe vegetation, and predominantly sandy soils with low organic matter content [32,33]. In contrast, the future centroids converge toward the Ordos Plateau and the Hetao Plain (Bayannur region), where the climate is semi-arid with mean annual precipitation of 200–400 mm, and the vegetation transitions to typical steppe and shrubland [34,35]. The eastward migration from the arid Alxa Plateau to the semi-arid Ordos–Hetao region reflects the species’ tracking of more favorable moisture and soil conditions. This limited latitudinal fluctuation, with all future centroids positioned slightly southward, suggests a strong latitudinal constraint on the distribution core of *C. korshinskii*, likely reflecting the concentration of optimal temperature and edaphic conditions (e.g., high base saturation soils on the Loess Plateau) within this mid-latitude zone. The slight southward tendency across all scenarios may be attributed to more pronounced warming at higher latitudes reducing suitability along the northern margins, while the southern margins retain favorable soil properties that buffer against climatic warming. This indicates that under the macro context of climate warming, the distribution core of *C. korshinskii* is deviating from the geometric center of its traditional range, consolidating in the semi-arid Ordos–Bayannur region.

## 3. Discussion

### 3.1. Physiological Mechanisms Underlying the Soil Preference of Caragana korshinskii

This study determined through the Biomod2 ensemble model that soil base saturation (t-bs) and soil moisture (soilmoisture) were consistently the two variables with the highest contribution rates across all scenarios, together explaining more than 38% of the model variance, indicating the dominant explanatory power of soil properties for the suitable distribution pattern of *C. korshinskii*. As a typical calcicole indicator plant, the pronounced preference of *C. korshinskii* for soils with high base saturation (optimal range 85–95%) has a clear physiological basis: calcium ions are not only a key cofactor for nitrogenase in rhizobia, with adequate Ca^2+^ supply being a necessary prerequisite for maintaining high nitrogen-fixing activity [27]; moreover, the neutral to slightly alkaline environment maintained by high-base-saturation soils effectively reduces aluminum and manganese toxicity [28]. Microbiological evidence further supports the primacy of soil properties in governing the Caragana-rhizobia symbiosis. Lu et al. (2009) demonstrated that soil salinity was a more important determinant of rhizobial community composition and geographic distribution across the Loess Plateau than climatic factors, indicating that edaphic conditions play a dominant role in structuring the symbiotic relationships of *C. korshinskii* [29]. This finding is consistent with our model results, in which soil base saturation and soil moisture consistently outranked climatic variables in explaining habitat suitability. We therefore propose as a working hypothesis that the high base saturation preference of *C. korshinskii* is mediated, at least in part, by enhanced symbiotic nitrogen fixation under favorable soil ionic conditions, although direct in situ measurements of nitrogenase activity along a base saturation gradient are still needed to test this mechanism definitively. The response curve of soil moisture exhibited a left-skewed unimodal pattern, with an optimal range of approximately 15–25%, and a sharp decline when exceeding 35%, revealing the pronounced physiological intolerance of *C. korshinskii* to excessive soil moisture, and also corroborating its conservative isohydric regulation behavior from a biogeographic perspective; long-term soil waterlogging can induce root hypoxia and increase disease risk [16]. Therefore, in some future regions, even if annual precipitation increases, if poor drainage leads to persistently high soil moisture, the suitability for *C. korshinskii* may decline rather than increase.

Altitude (alt) ranked third in contribution (15.0–17.5%), with its response curve declining continuously above 1500 m and approaching zero at 2500 m. This threshold likely reflects the constraints of low temperature on growing season length and the inhibitory effects of intense high-altitude radiation and freeze–thaw alternation on seedling establishment [30]. Under climate warming, warming at lower elevation zones can alleviate heat limitations to some extent, providing a mechanistic explanation for why the distribution centroid of *C. korshinskii* did not shift to higher elevations but migrated eastward with a minor southward fluctuation toward lower-elevation, high-latitude regions. The response curve for precipitation of the wettest month (bio13) was relatively flat across all scenarios, without a distinct optimal peak, suggesting that *C. korshinskii* has a wide tolerance range for single-month extreme precipitation intensity; as long as the soil has good drainage capacity, short-term heavy precipitation events are unlikely to cause direct stress. However, this also highlights the importance of soil drainage performance as a mediating factor—under the trend of intensifying precipitation extremes, the spatial heterogeneity of soil texture may become a key variable determining the local persistence of *C. korshinskii* [31]. The correspondence between suitability classes and soil variable ranges further supports this mechanistic interpretation. High suitability areas predominantly occur where soil base saturation exceeds 85% and soil moisture falls within the 15–25% optimal range, conditions characteristic of the loess deposits on the Ordos and Loess Plateaus. Moderate suitability areas correspond to regions where one of these two critical factors deviates from its optimum—either base saturation drops to 60–85% or soil moisture falls below 10% or exceeds 25%. Low suitability zones are characterized by both low base saturation and suboptimal moisture levels, typical of the arid Alxa Plateau margins and the sandy desert fringes.

The physiological mechanisms underlying these threshold responses are multifaceted. The high base saturation requirement reflects not only the calcium demand of rhizobial nitrogenase [27] but also the necessity of a stable pH buffering system for maintaining effective symbiosis, as the rhizobial strains associated with *Caragana* species are sensitive to acidic conditions [29]. The sharp decline in suitability when soil moisture exceeds 35% is attributed to the species’ conservative isohydric strategy, which, while adaptive under water deficit, becomes maladaptive under waterlogged conditions by restricting transpiration and nutrient uptake [16]. Importantly, soil moisture and base saturation interact synergistically: well-aerated, high base saturation soils simultaneously provide optimal conditions for root respiration, rhizobial nitrogen fixation, and nutrient diffusion, whereas excessive moisture disrupts these processes even when base status is favorable. This synergistic interaction explains why soil properties, rather than any single climatic factor, are the dominant determinants of *C. korshinskii* distribution. Taken together, the distribution of *C. korshinskii* is not driven by a single climatic or soil variable, but is controlled by a multi-factor synergistic constraint system with base saturation and water availability at the core, supplemented by topography and extreme precipitation.

### 3.2. Dynamics of the Distribution Change in Caragana korshinskii

Under the current climate scenario, the potential suitable habitat of *C. korshinskii* is mainly centered on the Ordos Plateau and the Loess Plateau, extending northeastward to the eastern Hetao Plain south of the Yin Mountains, and southwestward covering the southern margin of the Alxa Plateau and entering the eastern Hexi Corridor. This spatial pattern is highly consistent with the documented actual distribution range, indicating that the predicted results of the ensemble model have high geographic reliability [36,37]. Under future climate scenarios, the net loss of suitable habitat of *C. korshinskii* did not occur uniformly, but was concentrated as massive contraction along the southwestern and northwestern edges, with limited expansion at the northeastern end. This pronounced asymmetry between contraction and expansion was prominent across all scenarios, particularly under SSP585, where the ratio reached 2.42, revealing a severe situation where the rate of range margin retreat far exceeds the rate of new habitat colonization. Similar phenomena of edge retreat outpacing frontier expansion have been reported globally, such as the responses of alpine plants in the Iberian Peninsula and coniferous forests in North America to climate warming [38,39].

The causes of the above asymmetric response can be attributed to two aspects. First, the mismatch between climate velocity and species dispersal capacity. Loarie et al. (2009) estimated the median global climate velocity in flat areas to be approximately 0.42 km/year, and this rate may be higher in the arid interior of Asia due to faster warming [40]. As a shrub, the seeds of *C. korshinskii* are primarily dispersed by gravity, rodent transport, or surface runoff over short distances, lacking long-distance dispersal mechanisms; its natural migration capacity is far insufficient to track such rapid climatic spatial displacement [41]. Second, the spatial decoupling between substrate conditions and climatic suitability. Even if the climate in expansion areas (e.g., the northeast) becomes suitable in the future, effective population establishment may be hindered by the lack of calcareous soils with high base saturation. This phenomenon of “climatically suitable but edaphically unsuitable” suggests that species distribution models that do not consider soil substrate constraints are likely to overestimate the actual migration potential of species. Figueiredo et al. (2018), using Amazonian plants as an example, similarly emphasized the independent role of soil variables in regulating species range limits; even in regions with suitable climatic conditions, mismatched soil properties can still constitute distribution boundaries [31].

Under future climate change scenarios, in the contracting areas, *C. korshinskii* often exists as a constructive or dominant species. Its loss not only implies the disappearance of a single species but is more likely to trigger a series of cascading effects, including simplification of community structure, weakening of shrub sand-fixation functions, and collapse of the symbiotic nitrogen-fixation system, ultimately leading to an overall decline in regional ecosystem services [14]. Field observations on the Loess Plateau have directly documented such cascading degradation: declining *C. korshinskii* stands exhibit significantly lower soil organic carbon and total nitrogen, accelerated wind erosion, and diminished sand-fixation capacity relative to healthy stands [42]. A chrono sequence study further demonstrated that aging *C. korshinskii* plantations (>30 years) undergo shrub cover reduction and soil nutrient depletion, leading to the progressive loss of soil conservation functions [14]. These empirical findings provide direct support for the projected ecosystem degradation in contracting habitat zones. Particularly in ecologically fragile areas such as the western Loess Plateau, *C. korshinskii* stands serve as critical biological barriers for dune stabilization and soil conservation; once large-scale retreat occurs, it will directly threaten the ecological security and production base of the downwind agricultural-pastoral ecotone [42]. Therefore, the distribution range of contraction areas can serve as an effective early warning indicator for desertification in arid zones, providing spatially explicit scientific evidence for timely adaptive intervention measures.

### 3.3. Stable Habitats and Conservation Planning

The convergence of centroids toward the Ordos–Bayannur region identifies this area as the long-term stable core zone for *C. korshinskii* under future climate conditions. *C. korshinskii* possesses multiple inherent ecological advantages that make it particularly suitable for sustainable vegetation restoration. It has a deep root system and a conservative isohydric water-use strategy that allow survival under chronic water deficit [16]. Its symbiotic nitrogen-fixing capacity with rhizobia enhances soil fertility and facilitates the establishment of other native species [15,29]. It maintains photosynthetic activity and carbon assimilation even under severe water stress [13,42], and its proven windbreak and sand-fixation functions directly contribute to regional ecological security [14]. These traits collectively make *C. korshinskii* an ecologically sound and sustainable choice for long-term protective afforestation in this region. Based on our findings, priority should be given to promoting more resilient native shrubs such as *C. korshinskii* in this region to ensure long-term protective benefits. At the same time, it is recommended to establish a germplasm repository for *C. korshinskii* within the stable zone, collecting the genetic diversity of populations at the range margins to provide material for future assisted migration [43,44].

For contracting areas, proactive interventions should be implemented, such as artificial re-seeding, micro-catchment land preparation, and soil improvement, to slow the retreat process. In areas where reversal is truly impossible, gradual replacement with more drought- or barren-tolerant native herbaceous or sub-shrub species can be considered to maintain land cover and ecological functions. Although assisted migration carries certain ecological risks, small-scale trials can be conducted at suitable sites north of the core stable zone on the basis of rigorous genetic zoning and experimentation [45].

### 3.4. Limitations and Prospects

Based on the Biomod2 ensemble model and multi-source environmental data, this study comprehensively assessed the future trends of the potential suitable habitat of *C. korshinskii*. The ensemble modeling strategy employed a TSS-weighted committee averaging method to integrate predictions from the eight individual algorithms, effectively reducing the prediction uncertainty inherent in any single algorithm. By averaging across models with diverse algorithmic structures and weighting each by its validated predictive performance (TSS), the ensemble approach cancels out individual model biases and yields more robust consensus projections than any single model alone [21,22], and the simulated current distribution pattern was highly consistent with the documented range, indicating that the model has good spatial reliability and can provide a reference for climate change risk assessment of arid-zone shrubs [31,36]. This study still has limitations: first, the model did not include biological factors such as interspecific competition, grazing, pests, and diseases, which may significantly affect the establishment and survival of *C. korshinskii* at local scales [46]. Second, future distribution was extrapolated based only on current occurrence points, assuming niche conservatism; however, *C. korshinskii* populations may exhibit local adaptation, and different genotypes may have differentiated niches. Third, comprehensive multi-scalar drought indices such as the Standardized Precipitation Evapotranspiration Index (SPEI) were not incorporated. SPEI integrates precipitation and temperature to capture drought intensity, duration, and cumulative effects at multiple timescales [33], whereas our variable set (including AI and et0) primarily reflects long-term moisture availability and may not fully represent short-term drought dynamics that could critically affect the establishment and survival of *C. korshinskii*. Future studies could benefit from incorporating such multi-scalar drought indices. Fourth, human activity-related variables such as land use, population density, and grazing intensity were not considered in our models. Human activities are known to significantly influence species distributions in arid regions through habitat conversion, overgrazing, and afforestation practices [47]. Their exclusion means that our projections represent the potential climatic–edaphic envelope of *C. korshinskii* rather than its actual realized distribution under anthropogenic constraints. Future studies integrating spatially explicit land-use change scenarios alongside climate projections would allow a more comprehensive assessment of how natural and anthropogenic factors jointly determine the future distribution of this shrub. Integrative distribution models combining functional traits and genetic data would have greater explanatory power [48].

## 4. Materials and Methods

### 4.1. Species Occurrence Data

**Figure 8:**
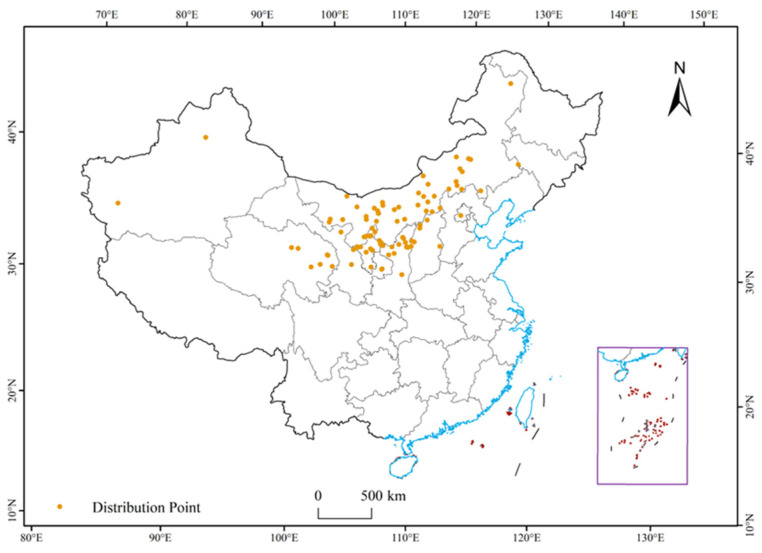
Species distribution of *Caragana korshinskii* in China. Note: This figure is based on the National Basic Geographic Information Center (http://bzdt.ch.mnr.gov.cn). The standard map with the approval number GS [2023] 2767 that was downloaded was created without any modifications to the base map. Same below.

Occurrence records of *C. korshinskii* were obtained from the Chinese Virtual Herbarium (CVH), the Global Biodiversity Information Facility (GBIF), and the published field survey literature. Because data from different sources (field surveys, databases, literature) may exhibit spatial clustering (i.e., multiple records in adjacent locations), which can induce spatial autocorrelation in SDMs, leading to overfitting and reduced predictive accuracy [49], this study applied the R package ENMTools to perform spatial thinning of occurrence data [50]. Specifically, only one unique occurrence point was retained within a 5 km buffer radius. This threshold is a commonly used distance for spatial thinning in species distribution modeling, which matches the spatial resolution of our environmental predictors (~1 km^2^ at 30 arc-seconds) and has been widely adopted in similar studies, effectively removing pseudo-replicates caused by georeferencing errors or clustered sampling while retaining sufficient occurrences for robust model training [51,52]. This processing effectively reduced the influence of spatial autocorrelation and significantly improved the accuracy of the model in predicting the potential geographic distribution of *C. korshinskii*. Ultimately, 118 valid occurrence points with longitude and latitude data were obtained. The processed occurrence data were imported into Excel and saved as a CSV file for subsequent model construction (Figure 8).

### 4.2. Environmental Variables

**Figure 9:**
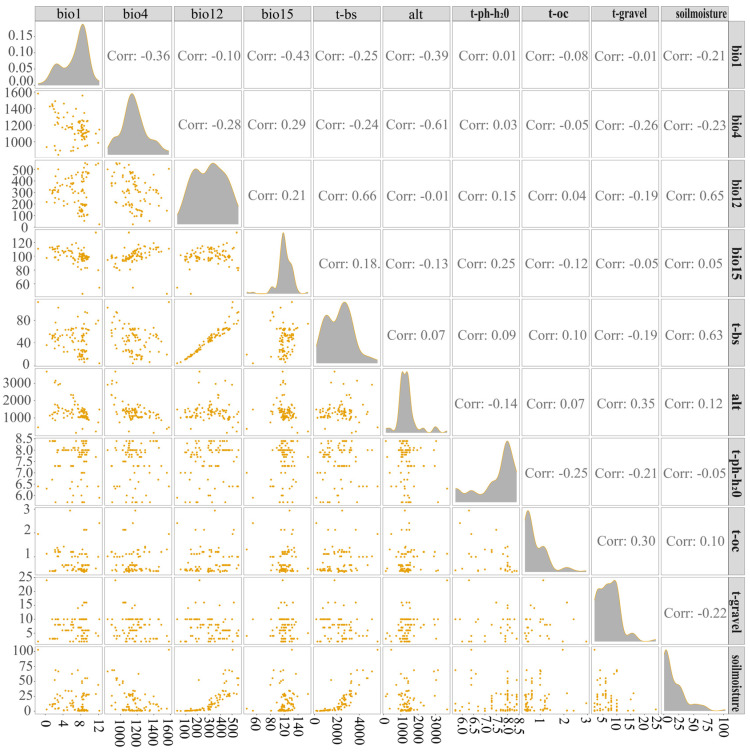
Correlation heatmap of modeling variables.

A total of 40 environmental factors were selected, including: 19 bioclimatic variables obtained from WorldClim 2.1 (current: 1970–2000; future: 2080–2100; resolution: 30 arc-seconds) under three SSP scenarios (SSP126, SSP370, SSP585) from the BCC-CSM2-MR model of CMIP6 [23,52]; 16 soil properties (soil moisture, organic carbon, pH, base saturation, electrical conductivity, texture, etc.) extracted from the HWSD soil database; topographic variables (altitude alt, slope, aspect) derived from the SRTM DEM; and drought indices (AI, aridity index; et0, potential evapotranspiration) obtained from CGIAR-CSI. Common comprehensive drought indices such as the Standardized Precipitation Evapotranspiration Index (SPEI) were not included because they are calculated from monthly temperature and precipitation data that are already represented among our 19 bioclimatic variables; including SPEI would introduce redundancy and increase the risk of multicollinearity. Instead, we relied on the Aridity Index (AI) and potential evapotranspiration (et0) to directly capture atmospheric water demand and long-term moisture availability. All variables were resampled to a uniform resolution of 30 arc-seconds (approximately 1 km) and constrained to the study extent (33–48° N, 97–118° E). The original spatial resolution of the environmental layers was as follows: bioclimatic variables (WorldClim 2.1), soil properties (HWSD), and drought indices (CGIAR-CSI) were at 30 arc-seconds; the SRTM digital elevation model was at 3 arc-seconds (approximately 90 m). Resampling of all continuous variables was performed using the bilinear interpolation method in ArcGIS 10.8 to preserve the continuous nature of the environmental gradients and ensure smooth transitions between adjacent grid cells. Through Pearson correlation analysis, for variable pairs with an absolute correlation coefficient greater than 0.7, only the variable with the higher contribution to the *C. korshinskii* distribution (based on preliminary model runs) was retained, while the redundant variable with the lower contribution was removed (Table 1, Figure 9) [53].

All species occurrence datasets, climate datasets, soil datasets, topographic DEM data and R software packages (R version 4.5.2) used in the above analysis were sourced from the publicly available databases listed in Table 2, where complete download URLs and access dates are provided to guarantee the repeatability of this study’s modeling results.

### 4.3. Ensemble Modeling and Evaluation

This study used the Biomod2 v4.2-1 platform (R 4.3.1) to integrate eight species distribution model algorithms: Classification Tree Analysis (CTA), Flexible Discriminant Analysis (FDA), Generalized Boosting Model (GBM), generalized linear model (GLM), Multivariate Adaptive Regression Splines (MARS), maximum entropy (MAXNET and MAXENT), and Random Forest (RF). MAXNET, which fits a MaxEnt model via a generalized linear model (GLM) framework, and the traditional MAXENT algorithm were run independently as two separate components to provide distinct realizations of the maximum entropy approach. Although both are based on the maximum entropy principle, MAXNET implements the model using the glmnet R package with L1 (lasso) regularization, which performs automatic feature selection through penalization [54], whereas MAXENT uses its own native optimization algorithm. Running both independently ensures that they contribute complementary information to the ensemble, thereby preventing redundant computation and enriching model diversity [21]. Model performance was evaluated using 4-fold cross-validation (80% of data for training, 20% for validation). Simulations under future climate scenarios used the same parameter configurations. All eight algorithms were run with their default parameters as provided in the Biomod2 package (version 4.2-1), with the following specified exceptions to ensure reproducibility [23]: for Random Forest (RF), the number of trees (ntree) was set to 500; for MaxEnt and MAXNET, only linear and quadratic feature classes were used and the maximum number of iterations was set to 500, following standard practice to avoid overly complex response curves [55,56]. The area under the receiver operating characteristic curve (AUC) and the true skill statistic (TSS) were used as quantitative indicators to assess model accuracy. An AUC value closer to 1 indicates a stronger ability of the model to discriminate between suitable and unsuitable areas: generally, 0.8 ≤ AUC < 0.9 is considered good, 0.9 ≤ AUC < 0.95 excellent, and AUC ≥ 0.95 outstanding [49]. TSS integrates the sensitivity and specificity of the model, with a value range of −1 to 1; values closer to 1 indicate higher predictive accuracy: generally, TSS > 0.4 is acceptable, 0.5 < TSS ≤ 0.7 good, 0.7 < TSS ≤ 0.85 excellent, and TSS > 0.85 outstanding [57]. Response curves for the dominant environmental variables were generated using the response. plot2 function in the Biomod2 package. This procedure produces univariate marginal response plots by holding all other environmental predictors constant at their median values and predicting habitat suitability across the observed range of each focal variable. The curves thus represent the isolated marginal effect of each variable on the ensemble model’s predicted occurrence probability, independent of correlations with other predictors. All response curves reflect the TSS-weighted ensemble consensus prediction. Based on these evaluation results, individual models were fused using a weighted average method with TSS values as weights to generate the ensemble model for the final prediction of the potential suitable distribution of *C. korshinskii*.

### 4.4. Suitability Classification and Analysis of Potential Distribution Dynamics

Based on the suitability probability values output by the Biomod2 ensemble model, this study adopted the Natural Breaks method (following the principle of minimizing intra-class variance and maximizing inter-class variance) [58] and used the spatial analysis tools in ArcGIS 10.8 for suitability classification. Combined with the actual distribution characteristics of *C. korshinskii*, the suitable areas were divided into four classes: unsuitable (0.00–0.10), low suitability (0.10–0.33), moderate suitability (0.33–0.64), and high suitability (0.64–1.00). The same classification criteria were applied to future scenarios. The Reclassify tool in ArcGIS was used to reclassify the raster data, and the area of each suitability class under different climate scenarios was calculated. Areas with a species occurrence probability > 0.10 (i.e., low, moderate, and high suitability zones) were defined as the potential distribution area, and their spatial distribution was mapped. The species probability maps (values ranging from 0, lowest probability, to 1, highest probability) obtained from the Biomod2 ensemble simulations under current and future climate conditions were imported into ArcGIS. Using the maximum training sensitivity plus specificity threshold as the criterion, the Con function was employed to convert the probability maps into 0/1 binary maps [50]. The SDMtoolbox 2.5 was used to partition the spatial change patterns of the suitable habitat of *C. korshinskii* by overlaying current and future binary suitability maps. Each grid cell was classified into one of three categories: Stable (predicted as suitable under both current and future conditions), Expansion (unsuitable under current conditions but suitable in the future), and Contraction (suitable under current conditions but unsuitable in the future). The binary maps were generated using the maximum training sensitivity plus specificity threshold, and to calculate the centroid positions of *C. korshinskii* under different climate backgrounds. The centroid shift trajectories relative to the current climate scenario were then mapped.

## 5. Conclusions

Using the Biomod2 ensemble modeling framework, this study systematically assessed the responses of the potential suitable habitat of *Caragana korshinskii* to climate change. The results showed that soil base saturation and soil moisture were the most important environmental drivers across scenarios, with their combined contribution exceeding that of the climatic variables. This highlights the critical role of soil properties in shaping the biogeography of calcicolous shrubs in arid regions. Under future climate, the suitable habitat of *C. korshinskii* is projected to decline continuously. Under the high-emission SSP585 scenario, total habitat loss approaches 10%, with highly suitable habitat experiencing particularly pronounced contraction. Across all scenarios, the habitat contraction exceeds expansion, and the contraction-to-expansion ratio increases with emission intensity, indicating an elevated risk of accelerated retreat at the species’ distribution margins. The distribution centroid of *C. korshinskii* is projected to shift eastward with a minor southward fluctuation. The Ordos–Bayannur region was identified as the long-term stable core habitat and should therefore be prioritized for germplasm conservation and ecological restoration. Meanwhile, proactive interventions are needed in the contracting zones along the southwestern and northwestern margins to mitigate potential losses of critical ecosystem services.

## Figures and Tables

**Table 2:** Detailed information of environmental datasets, species occurrence databases and R packages adopted for species distribution modeling.

No.	Full Name of Dataset	Abbreviation	Download URL
1	Chinese Virtual Herbarium	CVH	https://www.cvh.ac.cn/ (accessed on 5 May 2026)
2	Global Biodiversity Information Facility	GBIF	https://www.gbif.org/ (accessed on 5 May 2026)
3	R package ENMTools for spatial thinning	ENMTools	CRAN: https://cran.r-project.org/package=ENMTools (accessed on 9 May 2026) GitHub: https://github.com/danlwarren/ENMTools (accessed on 9 May 2026)
4	WorldClim 2.1 bioclimatic dataset	WorldClim 2.1	https://worldclim.org/data/worldclim21.html (accessed on 15 July 2025)
5	CMIP6 BCC-CSM2-MR climate model dataset	BCC-CSM2-MR (CMIP6)	https://esgf-data.dkrz.de/search/cmip6-dkrz.de/?mip_era=CMIP6&activity_id=ScenarioMIP&institution_id=BCC&source_id=BCC-CSM2-MR (accessed on 16 July 2025)
6	Harmonized World Soil Database v1.2	HWSD	https://www.fao.org/soils-portal/data-hub/soil-maps-and-databases/harmonized-world-soil-database-v12/en/ (accessed on 23 October 2025)
7	SRTM Digital Elevation Model	SRTM DEM	https://srtm.csi.cgiar.org/ (accessed on 17 July 2025)
8	CGIAR-CSI Global Aridity Index and Potential Evapotranspiration Database	CGIAR-CSI AI & ET0	https://cgiarcsi.community/data/global-aridity-and-pet-database/ (accessed on 15 July 2025)

## Data Availability

All environmental layers and species occurrence records used in this study were obtained from the publicly available datasets listed in Table 2, with complete official download links provided for each data source to ensure full data traceability and reproducibility of the modeling results.
